# Training benchmarks for the Fundamentals of Robotic Surgery virtual reality tasks

**DOI:** 10.1007/s00464-026-12957-5

**Published:** 2026-06-15

**Authors:** Noosha D. Deravi, Nathan Behrens, Brielle Warnock, Maya L. Hunt, Dimitrios Stefanidis, Amy Holmstrom, Katie Stanton Maxey, Samantha Tarras, E. Matthew Ritter

**Affiliations:** 1 Department of Surgery, Indiana University School of Medicine, Indianapolis, IN USA; 2 Department of Surgery, MedStar Georgetown University Hospital, Washington, DC USA

**Keywords:** Robotic surgery, Fundamentals of robotic surgery, Simulation-based training, Surgical education, Virtual reality, Curriculum

## Abstract

**Background:**

Given the recent acquisition of the Fundamentals of Robotic Surgery (FRS) by the Society of Gastrointestinal and Endoscopic Surgeons (SAGES), there is a need to develop a proficiency-based training paradigm for the console-based tasks. The purpose of this study was to establish defensible, expert-derived training benchmarks for the FRS virtual reality (VR) tasks.

**Methods:**

A known-group standard setting framework was utilized. Five fellowship-trained minimally invasive surgeons (> 250 robotic cases) performed one warm-up and two recorded repetitions of each FRS VR task on the da Vinci SimNow platform. Times to completion and total scores were aggregated to determine the measures of central tendency for training benchmarks. To establish validity evidence, first-attempt performance of novices (fourth-year medical students enrolled in a simulation-based elective) was compared to the expert-derived mean time and median total score using one-sample tests.

**Results:**

Puzzle Piece Dissection was the most time-consuming task among experts (300 ± 61 s) and novices (584 ± 181). Knot Tying was the lowest-scoring task and demonstrated the greatest variability among experts (median score 54, IQR = [0–89]), whereas Ring Tower Transfer was the lowest-scoring for novices (median score 1, IQR = [0–49]). The differences between novice performance and expert measures of central tendency were statistically discernible (*p* < 0.05*)* with a large effect size for all tasks. Thus, training benchmarks were set at less than or equal to the expert-derived trimmed mean time and greater than or equal to the expert-derived median score.

**Conclusion:**

Time- and score-based training benchmarks were established for the FRS VR tasks. The low and highly variable scores in Knot Tying were likely due to poor interaction fidelity, indicating that software modifications and/or alternative, non-VR exercises may be required for training. Further studies to evaluate the effectiveness of these training benchmarks are currently ongoing.

## Background

The Fundamentals of Robotic Surgery (FRS) program was initially developed to standardize a curriculum for and certification of robotic surgeons in response to a growing number of robotic-assisted surgeries [1]. Components of the curriculum were developed through multiple consensus conferences with experienced robotic surgeons from various professional societies and specialties. The psychomotor skills curriculum consists of six independent tasks, represented on a single dome-shaped platform, that address essential skills for robotic surgery. These skills and dome-based tasks include clutching, camera navigation, and wrist articulation (Ring Tower Transfer), suture handling and knot tying (Knot Tying), needle driving and suturing (Railroad Track), multi-arm control and cutting (Fourth Arm Cutting), atraumatic tissue handling and sharp dissection (Puzzle Piece Dissection), and energy sources and blunt dissection (Vessel Energy Dissection). Studies have demonstrated the effectiveness of utilizing these tasks for skills training [2].

**Fig. 1:**
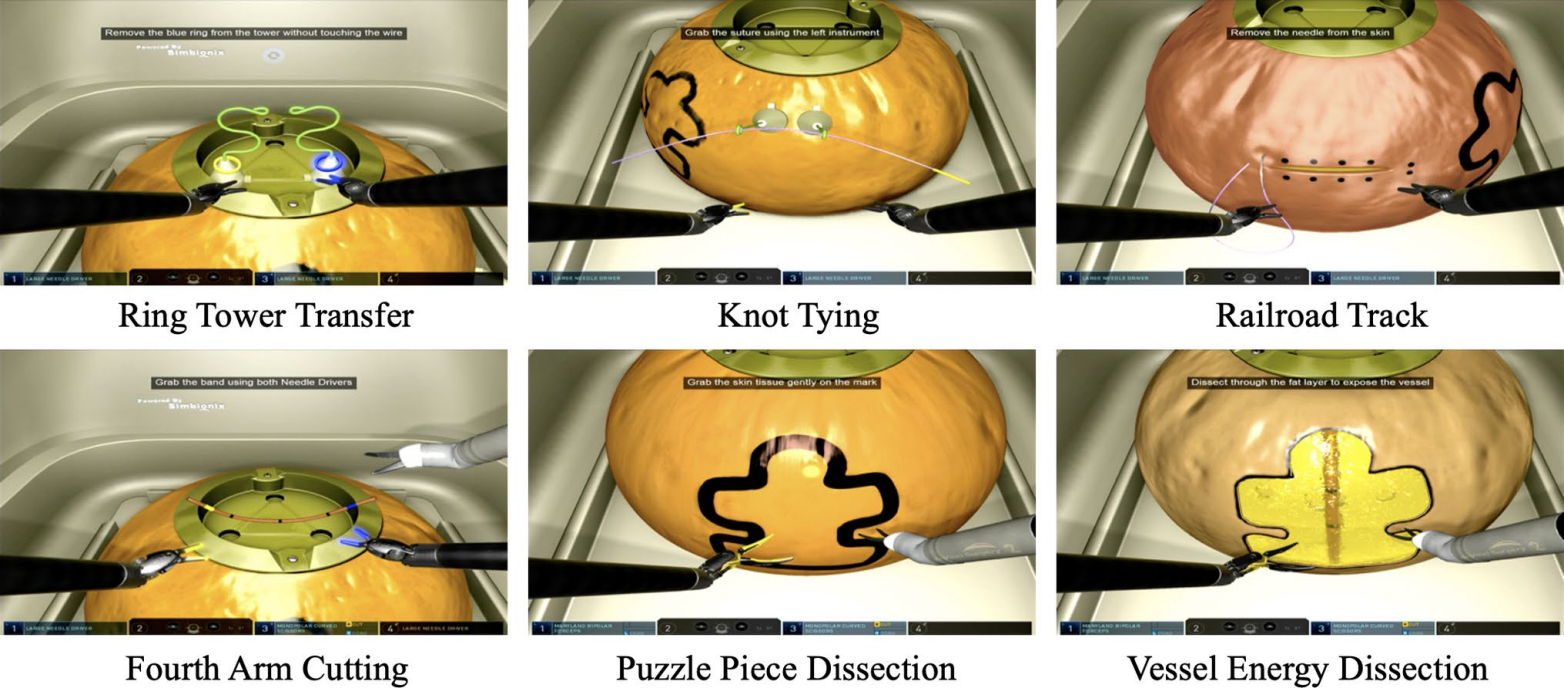
SimNow Fundamentals of Robotic Surgery virtual reality tasks

In 2025, FRS was acquired by the Society of Gastrointestinal and Endoscopic Surgeons (SAGES) with the intent of further developing the program in a way that parallels the Fundamentals of Laparoscopic Surgery (FLS) and the Fundamentals of Endoscopic Surgery (FES). Similar to these fundamental programs, it is important to develop a proficiency-based training paradigm that can be used by learners as they prepare for the psychomotor assessment. One potential, cost-effective option for training is with the virtual reality (VR) versions of the FRS tasks currently available through Surgical Science (Fig. 1) [3]. Prior studies have demonstrated the effectiveness of this VR training curriculum on technical skill acquisition for various platforms, including the da Vinci Simulation System, the Mimic dV-Trainer, and the RobotiX Mentor [2, 4]. However, there is currently no published proficiency-based curriculum for all six FRS tasks.

Additionally, most existing curricula that include the FRS VR tasks only utilize the simulator-generated total score—a combination of efficiency and penalty sub-totals—as the primary training benchmark [5–7]. From our group’s experience, the time component of the efficiency subtotal has been found to be nondiscriminating, with most individuals earning full points regardless of experience level or how quickly the task is performed. Given that task completion time discriminates well between different levels of robotic surgical skill, incorporating it into a proficiency-based curriculum for robotic surgery, similar to FLS, adds value [8, 9].

Thus, this study aimed to (1) establish expert-derived time- and score-based training benchmarks for all FRS VR tasks and (2) determine if those benchmarks are appropriate for a proficiency-based training curriculum by comparing novice baseline performance to these benchmarks.

## Methods

### Standard setting

To establish training benchmarks, a known-group standard setting framework was utilized as previously described [7]. Five experienced, fellowship-trained minimally invasive surgeons with greater than 250 robotic cases from a single academic institution served as the expert group. A threshold of 250 cases was chosen as it reflects a level of experience well beyond reported learning curves in robotic surgery [10]. For each task, experts performed one warm-up and two recorded repetitions on the Intuitive da Vinci Xi. Simulator-generated time to completion and total score were recorded. Total score was out of 100 and consisted of an efficiency subtotal score based on economy of motion and time, and a penalty subtotal score based on task-specific errors. Performance data from the recorded repetitions were aggregated to determine mean time to completion and median total score by task. The median was used for total score given skewed distributions and ceiling effects across multiple tasks. For mean time, attempts that resulted in a critical error and ended the task prematurely were excluded from calculations as the task was not performed to completion and would otherwise result in an inaccurately shortened time. Additionally, outliers greater than two standard deviations were removed to result in a trimmed mean time.

### Comparison of novice performance to expert-derived benchmarks

To demonstrate relationship to other variables validity evidence, novice performance was compared to the expert-derived training benchmarks. The rationale for this comparison is based on the notion that novice achievement of expert-level performance on the first attempt would suggest limitations in task design or benchmark leniency. Novices were fourth-year medical students enrolled in the Minimally Invasive Surgery for the Aspiring Surgeon course—a one-month, simulation-based elective for surgery-bound medical students interested in developing fundamental skills in laparoscopy, endoscopy, and robotic surgery. A total of 16 students (50% women, mean age 27)  participated in the course. Most students (86%, *n* = 14) had applied or were planning to apply to residency in general surgery, two in obstetrics and gynecology, one in urology, and one in anesthesia. The majority had no prior experience with the Legacy SimNow tasks; two students reported 1–5 h, and one reported 6–10 h. One student reported prior experience actively participating in a case from the surgeon console in the operating room. As part of an Introduction to Robotic Surgery coaching session, students viewed expert videos and observed a narrated in-person demonstration prior to attempting each task. Simulator-generated time to completion and total score were recorded.

For statistical analysis, time and total score from novices’ first attempts were compared to the expert-derived training benchmarks using one-sample *t* tests and one-sample Wilcoxon signed-rank tests, respectively. As with expert-derived mean times, any attempts that resulted in a critical error were excluded from calculations for time.

### Final training benchmarks

In alignment with proficiency-based training principles, benchmarks were anchored to expert performance to promote attainment of foundational skills at a level consistent with safe and reliable intraoperative performance. Training benchmarks for each task were defined as less than or equal to the expert-derived trimmed mean time, and greater than or equal to the expert-derived median score.

All statistical analyses were performed on SPSS Statistics, version 31 (IBM Corp., Arnock, NY). This study was part of a larger study that was reviewed by the Indiana University Institutional Review Board and deemed exempt.

## Results

### Time to completion

Puzzle Piece Dissection was the most time-consuming task among experts and novices (Table 1). Fourth Arm Cutting and Vessel Energy Dissection were the least time-consuming. All other tasks took between a mean of 71–135 seconds. For Knot Tying, four repetitions by experts and three attempts by novices ended prematurely due to a critical error secondary to tower avulsion and were excluded from time-related calculations. Mean total time to complete all tasks was 12 min (SD 2 min) for experts and 33 min (SD 9 min) for novices.

**Table 1:** Expert and novice first-attempt mean time to completion for the Fundamentals of Robotic Surgery virtual reality tasks

Task	Expert time in seconds, (SD)	Novice time in seconds, (SD)	*p-*value^a^	Effect size^b^ (Cohen’s *d*)
Ring Tower Transfer	92 (41)	386 (203)	< 0.001	1.5
Knot Tying	81 (29)	328 (206)	< 0.001	1.2
Railroad Track	135 (45)	476 (185)	< 0.001	1.8
Fourth Arm Cutting	31 (3)	90 (24)	< 0.001	2.4
Puzzle Piece Dissection	300 (61)	584 (181)	< 0.001	1.6
Vessel Energy Dissection	71 (19)	148 (54)	< 0.001	1.4

Expert repetitions *n* = 10, novice *n* = 16 (Knot Tying: expert *n* = 6, novice *n* = 13)

*SD* standard deviation

^a^*p-*value < 0.05 indicates statistically discernible differences between expert mean and novices’ first attempt times by one-sample *t* tests

^b^Cohen’s *d* > 0.8 indicates large effect size

Difference in time between novices’ first attempts and the expert-derived mean was statistically discernible (*p* < 0.05) with a large effect size (Cohen’s *d* > 0.80) for all tasks (Table 1).

### Total score

Knot Tying was the lowest-scoring task and demonstrated the greatest variability in performance among experts (Table 2). Four expert repetitions resulted in a score of zero (three of these attempts were from the second recorded repetition) due to a critical error secondary to tower avulsion while tying knots. Fourth Arm Cutting was the highest-scoring task for experts and demonstrated the least amount of variability in performance with four experts earning a score of 100 on every recorded repetition. Vessel Energy Dissection was the highest-scoring task for novices with a quarter earning a score of 100 on the first attempt.

**Table 2:** Expert and novice first-attempt median total score for the Fundamentals of Robotic Surgery virtual reality tasks

Task	Expert score^a^ (IQR)	Novice score^a^ (IQR)	*p-*value^b^	Effect size^c^ (*r*)
Ring Tower Transfer	94 (91–97)	1 (0–49)	< 0.001	0.9
Knot Tying	54 (0–89)	5 (0–48)	0.003	0.7
Railroad Track	95 (88–98)	4 (0–55)	< 0.001	0.9
Fourth Arm Cutting	100 (100–100)	86 (75–97)	< 0.001	0.8
Puzzle Piece Dissection	84 (78–92)	74 (46–82)	0.004	0.7
Vessel Energy Dissection	98 (88–98)	91 (77–100)	< 0.001	0.7

Expert repetitions *n* = 10, novice *n* = 16

*IQR* interquartile range

^a^Simulator-generated total score (max 100) = efficiency minus task-specific penalties

^b^*p*-value < 0.05 indicates statistically discernible differences between expert median and novices’ first attempt scores by one-sample Wilcoxon signed-rank tests

^c^*r* > 0.5 indicates large effect size

Differences in total score between novices’ first attempts and the expert-derived median were statistically discernible (*p* < 0.05) with a large effect size for all tasks (*r* > 0.08). However, scores for Puzzle Piece Dissection and Vessel Energy Dissection did overlap between novices and experts.

### Training benchmarks

Expert-derived training benchmarks are shown in Table 3. On the first attempt, one novice achieved the time *and* score benchmarks for Puzzle Piece Dissection, and two did so for Vessel Energy Dissection. None of these novices reported prior experience with the simulators.

**Table 3:** Expert-derived training benchmarks for the Fundamentals of Robotic Surgery virtual reality tasks

Task	Time (seconds)	Total score^a^
Ring Tower Transfer	≤ 92	≥ 94
Knot Tying	≤ 81	≥ 54
Railroad Track	≤ 135	≥ 95
Fourth Arm Cutting	≤ 31	100
Puzzle Piece Dissection	≤ 300	≥ 84
Vessel Energy Dissection	≤ 71	≥ 98

^a^Simulator-generated total score (max 100) = efficiency minus task-specific penalties

## Discussion

This study demonstrates the establishment of time- and score-based training benchmarks for the FRS VR tasks that effectively discriminate between novice learners and expert surgeons. The overlap in total scores between novices and experts for Fourth Arm Cutting, Puzzle Piece Dissection, and Vessel Energy Dissection underscores the importance of including secondary performance metrics, such as time to completion, as an additional benchmark. The score-based training benchmarks in this study for Fourth Arm Cutting and Puzzle Piece Dissection are similar to those described by Tellez et al., but direct comparisons are limited due to the use of different measures of central tendency [7].

Knot Tying demonstrated the lowest and most variable scores among experts with multiple attempts resulting in scores of zero. This was likely secondary to poor interaction fidelity as the VR suture does not exhibit the same behavioral properties as physical suture. The impact of this was primarily seen during formation of the surgeon’s knot. While most robotic surgeons perform this step by wrapping the suture around the instrument tip twice, attempting to do so in the VR version resulted in the suture consistently slipping off the instrument. Additionally, successfully formed knots were occasionally not registered by the simulator and required repeated attempts. This intermittently prompted experts to use nontraditional strategies to maximize simulator performance rather than techniques reflective of true operative practice. These findings suggest that further software refinement or non-VR training modalities may be required to ensure acquisition of appropriate suture management and knot tying techniques. Conversely, Fourth Arm Cutting appeared relatively easy and took the least amount of time for both experts and novices. Thus, perhaps a more challenging task is needed for training of multi-arm control and cutting. Given concerns regarding the simulator’s ability to adequately capture the underlying construct of robotic surgical skill, as well as potential threats to validity (i.e., construct underrepresentation and construct-irrelevant variance), the SAGES FRS Task Force is evaluating which additional tasks may benefit from redesign prior to formal implementation [11].

A limitation of this study relates to the collection of novice performance data. The relative ease of Vessel Energy Dissection with two novices achieving perfect scores on the first attempt may be attributable to coaching by instructors familiar with task-specific designs and assessment metrics set by the manufacturer. More broadly, three novices met both expert-derived time and score-based training benchmarks on their first attempts (one for Puzzle Piece Dissection; two for Vessel Energy Dissection), suggesting that successful performance of these VR tasks may not fully reflect the technical skills required for robotic surgery. Additionally, a quarter of novices reported some experience with the surgeon console, which may have contributed to higher scores than expected in this cohort. Consequently, the observed differences between expert and novice performance may underestimate the degree of baseline differences between these two groups.

It is important to note that these benchmarks represent one component of a proficiency-based training curriculum. Achieving proficiency on the simulator should also include requirements for repeated benchmark performance to ensure reliable skill acquisition and promote retention through spaced repetition. For example, the FLS curriculum requires two consecutive repetitions followed by ten non-consecutive repetitions for Peg Transfer and Intracorporeal Knot Tying [9]. Although no universal standard exists for defining proficiency based on the number and/or temporal spacing of repetitions, future definitions should be informed by evidence on both learning and retention [12, 13]. Furthermore, while achieving simulator-based proficiency can serve as a marker of initial skill development in robotic surgery, the impact of training to these specific benchmarks on intraoperative performance requires further investigation [14].

In this study, we demonstrated the establishment of expert-derived time- and score-based training benchmarks for all FRS VR tasks and provided some validity evidence for their discriminatory ability between novice learners and experienced surgeons. While some tasks will benefit from software modifications to improve functional task alignment or will require alternative, non-VR platforms, this study contributes to the development of an initial standardized training paradigm for the psychomotor skills component of the current FRS program. These benchmarks offer a cost-effective framework that can be readily implemented by other institutions with existing VR platforms. Future studies focused on evaluating the effectiveness of these training benchmarks and transferability to inanimate task performance are currently ongoing.
